# Supplementary material for: Diagnosing depression in primary care: a Rasch analysis of the Major Depression Inventory
Source: Scand J Prim Health Care. 2019 Apr 26;37(2):256–63. doi: 10.1080/02813432.2019.1608039 (PMC6567252; doi:10.1080/02813432.2019.1608039)

**Supplementary Data**

Table 1: Residual Correlation Matrix for all 10 MDI items


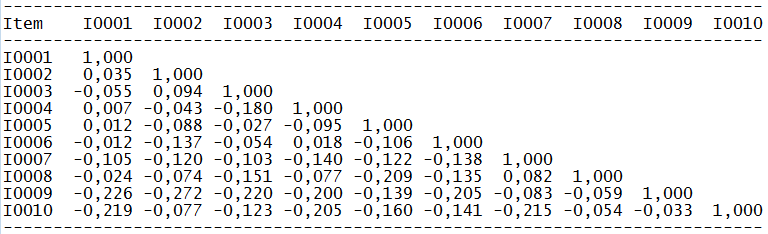


Table 2: Person Factor ANOVA for gender; 236 (65%) females and 127 (35%) males


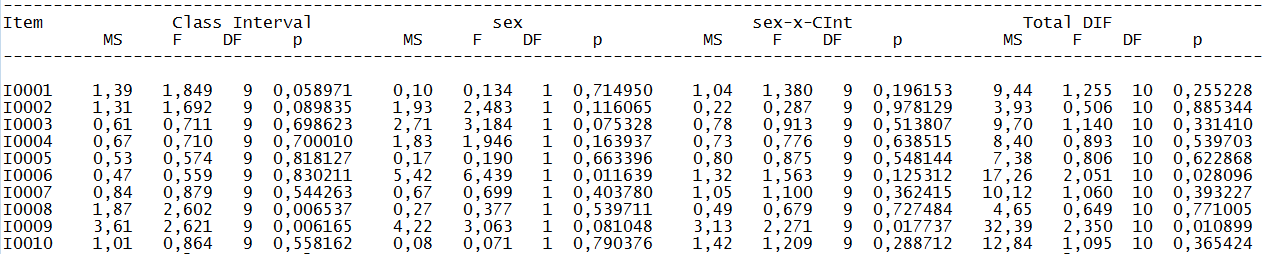


Table 3: Person Factor ANOVA for age group, dichotomized at median of 55 years (N= 363)


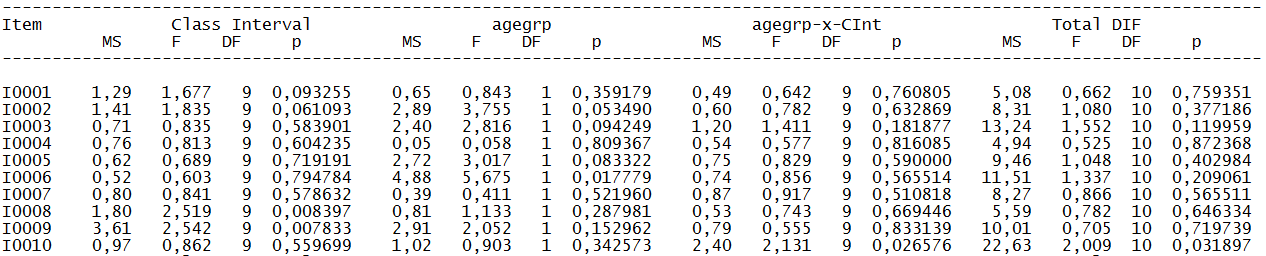

Supplement: Supplemental Material [file IPRI_A_1608039_SM9107.docx]
